# Reconstructed monthly river flows for Irish catchments 1766–2016

**DOI:** 10.1002/gdj3.107

**Published:** 2020-10-08

**Authors:** Paul O’Connor, Conor Murphy, Tom Matthews, Robert L. Wilby

**Affiliations:** ^1^ Irish Climate Analysis and Research Units Department of Geography Maynooth University Maynooth Co. Kildare Ireland; ^2^ Department of Geography and Environment Loughborough University Loughborough UK

**Keywords:** hydrological modelling, Ireland, reconstruction, river flow, time series

## Abstract

A 250‐year (1766–2016) archive of reconstructed river flows is presented for 51 catchments across Ireland. By leveraging meteorological data rescue efforts with gridded precipitation and temperature reconstructions, we develop monthly river flow reconstructions using the GR2M hydrological model and an Artificial Neural Network. Uncertainties in reconstructed flows associated with hydrological model structure and parameters are quantified. Reconstructions are evaluated by comparison with those derived from quality assured long‐term precipitation series for the period 1850–2000. Assessment of the reconstruction performance across all 51 catchments using metrics of MAE (9.3 mm/month; 13.3%), RMSE (12.6 mm/month; 18.0%) and mean bias (−1.16 mm/month; −1.7%), indicates good skill. Notable years with highest/lowest annual mean flows across all catchments were 1877/1855. Winter 2015/16 had the highest seasonal mean flows and summer 1826 the lowest, whereas autumn 1933 had notable low flows across most catchments. The reconstructed database will enable assessment of catchment specific responses to varying climatic conditions and extremes on annual, seasonal and monthly timescales.

## Graphical abstract

Long‐term river flow records are fundamental for understanding the risk posed by drought and high flow events. Whilst in many locations long‐term flow records are lacking, precipitation data rescue efforts have made extended climatic data readily available and when employed in tandem with hydrological models such data can be used to generate historic flows. Here, we use such an approach to generate a 250 year (1766–2016) monthly flow reconstruction archive for 51 catchments in Ireland.

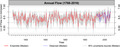

## 1 INTRODUCTION

Continuous, long‐term river flow records are needed for evaluations of hydro‐climatic variability and change, historical extremes and catchment processes (Machiwal and Jha, 2006). They also underpin water management and provide a means of stress‐testing existing and planned systems to a range of variability and past droughts (Wilby and Murphy, 2019). Unfortunately, there are few continuous and homogeneous river flow records spanning a century or more (Mediero *et al*., 2015). Instead, available records are often impacted by confounding factors or large amounts of missing data (Wilby *et al*., 2017).

Various techniques exist for extending observations by reconstructing river flows. This typically involves forcing statistical or conceptual hydrological models with long‐term precipitation and temperature/evapotranspiration data provided by reanalysis (e.g. Kuentz *et al*., 2013; Brigode *et al*., 2016) or long‐term historical data sets (e.g. Jones, 1984; Spraggs *et al*., 2015; Crooks and Kay, 2015; Rudd *et al*., 2017; Hanel *et al*., 2018; Smith *et al*., 2019; Noone and Murphy, 2020). Others have leveraged international data rescue initiatives to generate gridded historical weather variables (Casty *et al*., 2007). Whilst these kinds of information have been used to reconstruct river flows in parts of Europe (e.g. Moravec *et al*., 2019), they have yet to be deployed in the British‐Irish Isles.

Here, we develop a data set of reconstructed monthly river flows for 51 catchments across the island of Ireland back to 1766. This was achieved using gridded historical meteorological data, bias corrected to contemporary observations in each catchment. These data provided the input to a conceptual hydrological model and an artificial neural network (ANN), both of which were trained and verified using river flow observations. In addition, we use recently rescued precipitation data to evaluate model reconstructions for selected catchments during the period 1850–2010. The following sections describe the catchments, data sets and modelling approaches, before we present the derived reconstructions.

## 2 DATA PRODUCTION METHODS

### 2.1 Catchments and data

**FIGURE 1:**
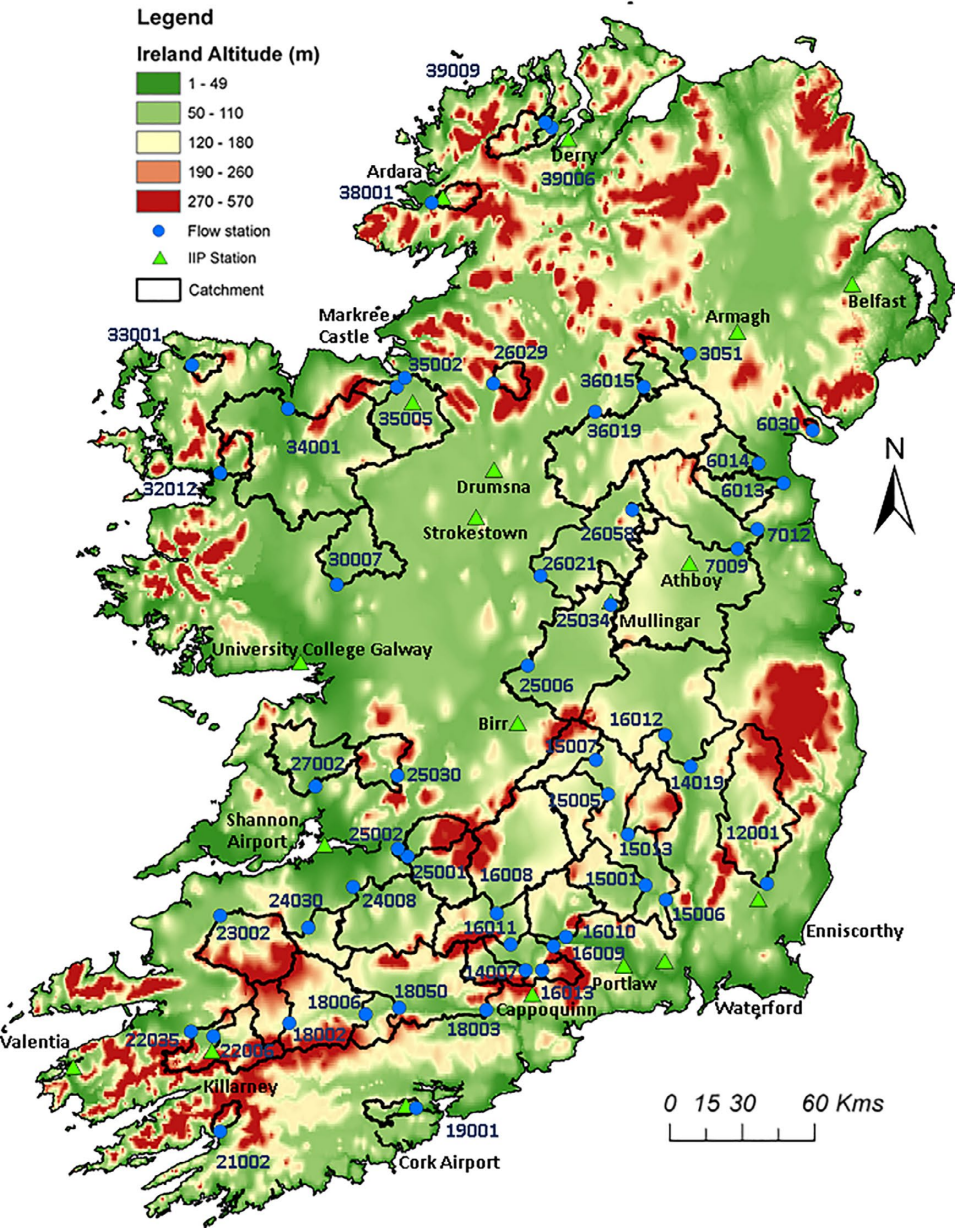
The 51 study catchments along with the locations of corresponding flow stations and island of Ireland precipitation (IIP) series synoptic stations

Reconstructions were generated for 51 catchments (Table 1 and Figure 1) that are relatively free from artificial influences (following criteria applied by Murphy *et al*. (2013): they have at least 25 years of record and acceptable quality rating curves). The catchments are broadly representative of hydro‐climatological conditions across the island, with a recognized under‐representation of upland catchments along coastal margins (Broderick *et al*., 2019). Urban extent averages <2% of the combined area of all catchments, which individually vary in size between 10 and 2,418 km^2^. However, given the extent of arterial drainage works undertaken in Ireland, it is unavoidable that some catchments have been impacted by such activities. We note which catchments are known to be affected by arterial drainage in Table 1.

**Table 1:** Details of the 51 catchments for which flow reconstructions were generated

River	Flow station	Waterbody	Arterial	Area	Calibration	GR2M validation scores			ANN validation scores			Ensemble validation scores		
ID	Name	Name	Drainage	km^2^	Years	NSE	KGE	PBIAS%	NSE	KGE	PBIAS%	NSE	KGE	PBIAS%
3051	Faulkland	Blackwater (Mon)	Yes	143	1976–2000	0.79	0.69	−16.40	0.78	0.67	−14.80	0.80	0.69	−16.00
6013	Charleville	Dee	Yes	309	1976–2000	0.78	0.75	−12.50	0.82	0.70	−9.10	0.83	0.72	−11.20
6014	Tallanstown	Glyde	Yes	270	1976–2000	0.77	0.75	−8.00	0.82	0.73	−4.50	0.81	0.73	−7.70
6030	Ballygoly	Big	No	10	1975–2000	0.86	0.86	−2.10	0.83	0.78	−1.00	0.86	0.83	−1.80
7009	Navan Weir	Boyne	Yes	1658	1977–2000	0.77	0.73	−9.90	0.81	0.73	−6.40	0.81	0.71	−9.60
7012	Slane Castle	Boyne	Yes	2,408	1961–2000	0.79	0.74	−11.90	0.83	0.71	−8.60	0.84	0.72	−10.90
12001	Scarrawalsh	Slaney	No	1,031	1961–2000	0.76	0.73	−15.10	0.80	0.81	−11.90	0.78	0.74	−14.50
14007	Derrybrock	Stradbally	No	115	1980–2000	0.83	0.77	−10.10	0.88	0.79	−8.80	0.86	0.75	−10.30
14019	Levitstown	Barrow	No	1697	1961–2000	0.81	0.74	−12.40	0.86	0.79	−8.00	0.84	0.74	−11.60
15001	Annamult	Kings	No	445	1972–2000	0.87	0.90	−1.00	0.88	0.82	1.10	0.89	0.87	−0.30
15003	Dinin Bridge	Dinin	No	140	1972–2000	0.87	0.86	−7.40	0.84	0.75	−8.90	0.88	0.82	−7.80
15005	Durrow Ft. Br.	Erkina	No	379	1972–2000	0.78	0.68	−11.90	0.83	0.72	−8.50	0.82	0.68	−10.60
15006	Brownsbarn	Nore	No	2,418	1972–2000	0.87	0.88	−3.90	0.90	0.87	0.30	0.91	0.87	−2.10
15007	Kilbricken	Nore	No	340	1982–2000	0.82	0.71	−11.30	0.81	0.68	−8.60	0.82	0.70	−10.70
16008	New Bridge	Suir	No	1,090	1961–2000	0.85	0.84	−6.90	0.86	0.87	−2.40	0.88	0.84	−5.40
16009	Caher Park	Suir	No	1583	1962–2000	0.86	0.87	−7.50	0.90	0.93	−3.20	0.89	0.89	−6.20
16010	Anner	Anner	No	437	1973–2000	0.80	0.89	−1.20	0.84	0.92	2.90	0.85	0.91	0.60
16011	Clonmel	Suir	No	2,144	1962–2000	0.88	0.89	−0.10	0.89	0.94	3.20	0.90	0.90	1.10
16012	Tar Bridge	Tar	No	230	1969–2000	0.83	0.83	0.80	0.83	0.89	2.00	0.85	0.85	1.30
16013	Fourmilewater	Nire	No	94	1973–2000	0.69	0.85	−0.80	0.82	0.84	−1.20	0.80	0.86	−1.50
18002	Ballyduff	Blackwater	No	2,334	1972–2000	0.90	0.91	6.00	0.87	0.86	10.30	0.91	0.92	7.40
18003	Killavullen	Blackwater	No	1,257	1972–2000	0.91	0.88	0.80	0.92	0.95	3.80	0.93	0.91	1.60
18006	Cset Mallow	Blackwater	No	1,052	1978–2000	0.88	0.83	−0.20	0.89	0.88	3.80	0.90	0.85	1.10
18050	Duarrigle	Blackwater	No	250	1982–2000	0.89	0.86	−5.50	0.89	0.84	−5.00	0.90	0.85	−5.30
19001	Ballea	Owenboy	No	103	1973–2000	0.85	0.76	−16.30	0.82	0.74	−12.60	0.85	0.75	−14.50
21002	Coomhola	Coomhola	No	65	1976–2000	0.90	0.83	−6.40	0.92	0.89	−4.70	0.93	0.87	−5.30
22006	Flesk	Flesk (Laune)	No	329	1990–2000	0.84	0.75	−7.20	0.91	0.84	−6.40	0.89	0.80	−6.60
22035	Laune Bridge	Laune	Yes	560	1992–2000	0.81	0.73	−6.10	0.87	0.84	−6.30	0.86	0.78	−6.40
23002	Listowel	Feale	Yes	647	1975–2000	0.93	0.90	2.80	0.92	0.87	3.40	0.94	0.89	3.40
24008	Castleroberts	Maigue	Yes	806	1977–2000	0.89	0.83	−0.40	0.88	0.83	3.30	0.90	0.82	0.90
24030	Danganbeg	Deel	Yes	259	1981–2000	0.92	0.90	1.70	0.91	0.90	4.20	0.93	0.90	2.80
25001	Annacotty	Mulkear	Yes	648	1973–2000	0.88	0.82	−1.50	0.88	0.82	0.00	0.89	0.82	−1.00
25002	Barrington Br.	Newport (Mun)	Yes	230	1961–2000	0.91	0.92	−0.90	0.91	0.94	0.70	0.92	0.93	−0.20
25006	Ferbane	Brosna	No	1,163	1961–2000	0.83	0.79	−10.40	0.86	0.80	−6.80	0.87	0.78	−9.50
25030	Scarriff	Graney	Yes	279	1973–2000	0.86	0.81	−6.80	0.86	0.83	−4.10	0.87	0.82	−5.50
25034	Rochfort	L. Ennell Trib	Yes	11	1976–2000	0.81	0.84	−7.90	0.85	0.81	−7.60	0.86	0.82	−8.30
26021	Ballymahon	Inny	No	1,099	1973–2000	0.83	0.86	−4.10	0.84	0.88	−0.60	0.88	0.85	−3.40
26029	Dowra	Shannon	Yes	117	1976–2000	0.86	0.85	−8.80	0.83	0.81	−8.00	0.86	0.84	−8.20
26058	Ballyrink Br.	Inny Upper	Yes	60	1982–2000	0.75	0.82	4.50	0.85	0.82	4.40	0.85	0.82	2.60
27002	Ballycorey	Fergus	Yes	511	1961–2000	0.83	0.74	−8.70	0.84	0.81	−6.90	0.85	0.75	−8.50
30007	Ballygaddy	Clare	No	470	1975–2000	0.89	0.85	−7.50	0.91	0.90	−4.30	0.91	0.86	−6.40
32012	Newport Weir	Newport	No	146	1982–2000	0.90	0.85	−6.00	0.90	0.89	−3.80	0.91	0.87	−4.80
33001	Glenamoy	Glenamoy	Yes	76	1978–2000	0.93	0.95	−0.50	0.87	0.94	0.00	0.93	0.96	0.30
34001	Rahans	Moy	No	1975	1970–2000	0.90	0.92	−4.60	0.89	0.94	−1.90	0.92	0.92	−4.00
35002	Billa Bridge	Owenbeg	No	81	1972–2000	0.86	0.88	4.70	0.87	0.92	5.60	0.88	0.90	5.30
35005	Ballysadare	Ballysadare	No	640	1961–2000	0.89	0.90	−2.30	0.90	0.90	−2.10	0.91	0.90	−2.40
36015	Anlore	Finn	No	153	1973–2000	0.84	0.76	−9.50	0.79	0.65	−10.40	0.84	0.73	−9.80
36019	Belturbet	Erne	No	1,492	1961–2000	0.83	0.85	−5.60	0.79	0.77	−7.60	0.86	0.80	−6.90
38001	Clonconwal	Ownea	No	111	1973–2000	0.93	0.87	−4.00	0.93	0.88	−4.50	0.94	0.88	−3.60
39006	Lennan	Claragh	No	245	1977–2000	0.85	0.88	8.00	0.85	0.87	7.90	0.86	0.88	8.00
39009	Aghawoney	Fern O/L	Yes	207	1973–2000	0.91	0.90	−1.00	0.91	0.88	−2.20	0.92	0.90	−1.20

Note

Included are calibration periods for each catchment, together with logNSE, KGE and PBIAS scores for the validation period (2001–2016) for ANN, GR2M and Ensemble median simulations.

Daily flow series were obtained from the Office of Public Works (OPW; http://waterlevel.ie/) and the Environmental Protection Agency (http://www.epa.ie/hydronet/) and then aggregated to monthly mean flows. The average amount of missing data was <6% across the 51 catchments, with a notable outlier of 31% being the Blackwater at Duarrigle (ID: 18050). Of the total missing days (11% overall), the majority have been previously infilled using rainfall–runoff modelling techniques (Murphy *et al*., 2013). As the remaining missing data only represented 1% of the total, they were not repopulated.

We use gridded (1 × 1 km) monthly precipitation and temperature series (Walsh, 2012) area‐averaged for each catchment, alongside concurrent river flow records, to calibrate the hydrological models (see below). Monthly potential evapotranspiration (PET) was estimated from air temperature and radiation following the method of Oudin *et al*. (2005). We favoured this over more physically based methods (e.g. Penman‐Monteith), because the latter have greater data requirements (e.g. wind speed, humidity) that cannot be met over the full duration of the reconstruction period. Instead, the sensitivity of monthly river flow simulations to PET estimation methods was tested for periods with complete variable sets. Six PET estimation methods (Penman–Monteith Penman (1948), Monteith (1965), Blaney and Criddle (1950), Hamon (1961), Oudin *et al*. (2005), Thornthwaite (1948) and Kharrufa (1985)) were evaluated using the hydrological model GR2M. This revealed that the Oudin method performed similarly to the Penman–Monteith method, with an average RMSE of 3.6 mm between flows generated from the two methodologies for five catchments for the period 1974–2000 (equating to 4.5% of mean annual flows).

### 2.2 Historical gridded precipitation and temperature data

**FIGURE 2:**
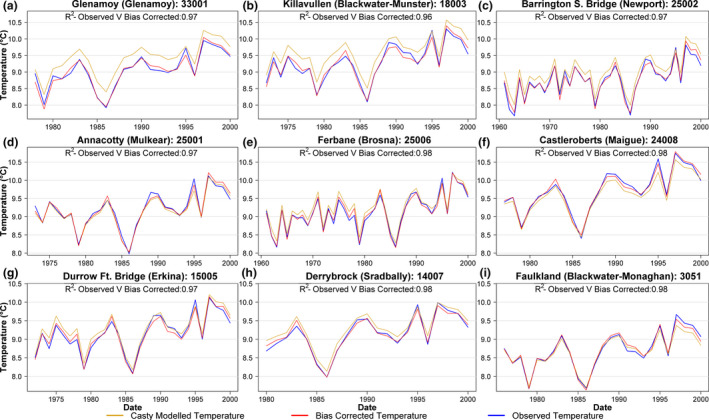
Annual bias corrected Casty temperature for nine catchments from the start of the respective observations up until the year 2000. *R* ^2^ scores between bias corrected and observed temperature values are also provided

**FIGURE 3:**
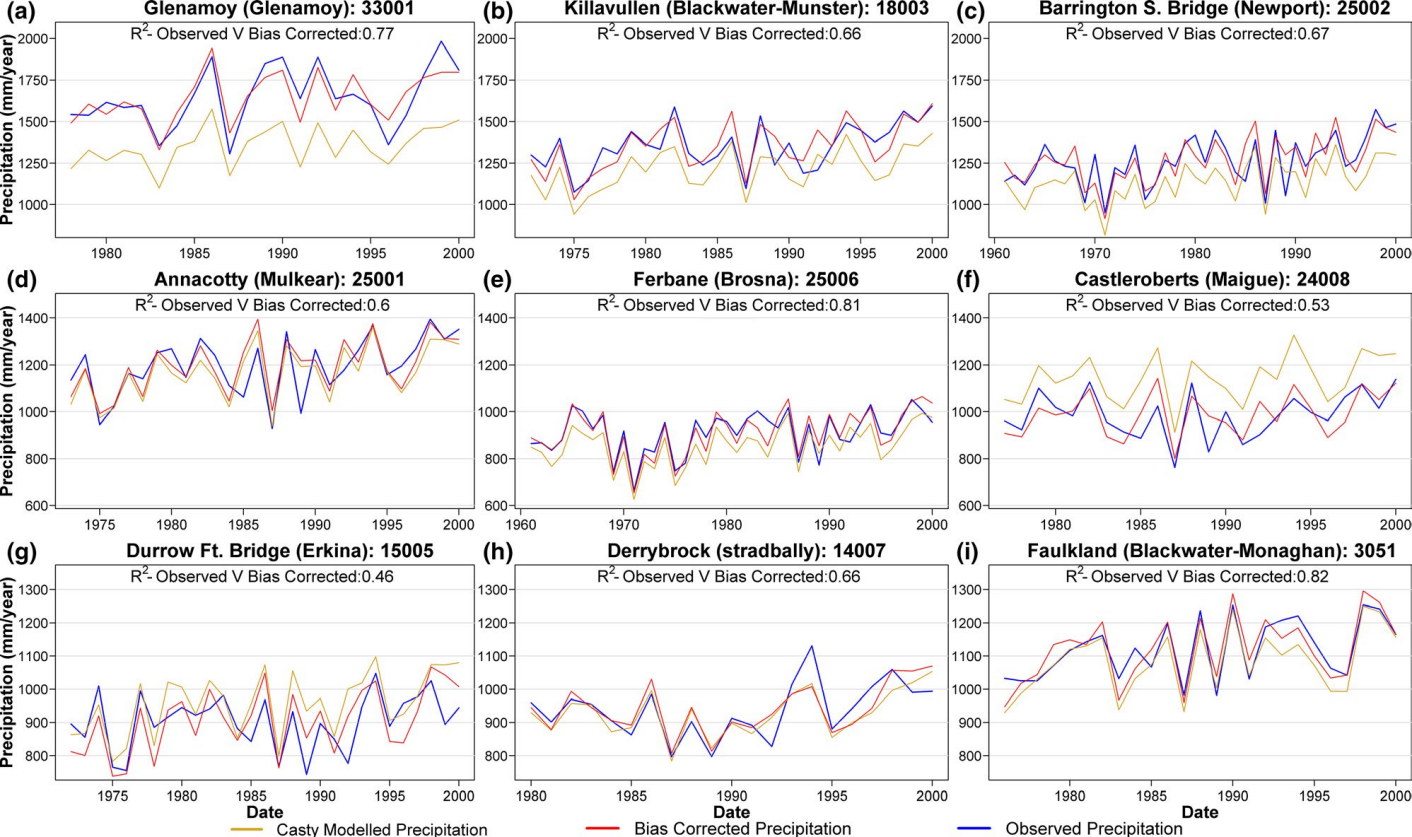
Annual bias corrected Casty precipitation values for nine catchments from the start of the respective observations up until the year 2000. *R* ^2^ scores between bias corrected and observed precipitation values are also provided

Casty *et al*. (2007) (henceforth Casty data) produced gridded (0.5° × 0.5°) monthly temperature and precipitation series for Europe covering the period 1766–2000 using non‐linear principle component regression of a spatial network of available station data against reanalysis data, with independent predictors used for different variables (Casty *et al*., 2007). Monthly mean temperature and total precipitation were extracted and averaged for grids overlying each catchment for the years 1766–2000. Quantile mapping (Maraun, 2016) was used to bias correct Casty data to catchment averages using the aforementioned gridded (1 × 1 km) monthly precipitation and temperature series. We perform quantile mapping by interpolating the empirical quantiles using local linear least square regression to robustly estimate the values of the quantile–quantile relationship between the Casty and observed data for each catchment. For values outside the historical range, a constant correction—equivalent to the highest quantile in that series—was applied (Boé *et al*., 2007). Bias correction was carried out on a monthly basis using the ‘qmap’ R package (Gudmundsson, 2016). Sample bias correction plots for nine catchments are shown in Figure 2 (temperature) and Figure 3 (precipitation). Across the 51 catchments, the bias adjustment produced minimal change in mean annual temperature values (−0.15°C). Precipitation corrections were more substantial, with a mean increase of 94.2 mm/year (7.7% of mean annual precipitation). Once bias corrected, observed temperature and precipitation were appended to each catchment series to bring values up to 2016. The Oudin method was then used to derive PET estimates from the Casty temperature data for each catchment.

### 2.3 Hydrological models and calibration procedures

To ascertain the contribution to uncertainty generated by model structure, two model types were implemented—a conceptual hydrological model (GR2M) and an empirical based Artificial Neural Network (ANN). These models are explained below.

#### 2.3.1 The GR2M conceptual model

**FIGURE 4:**
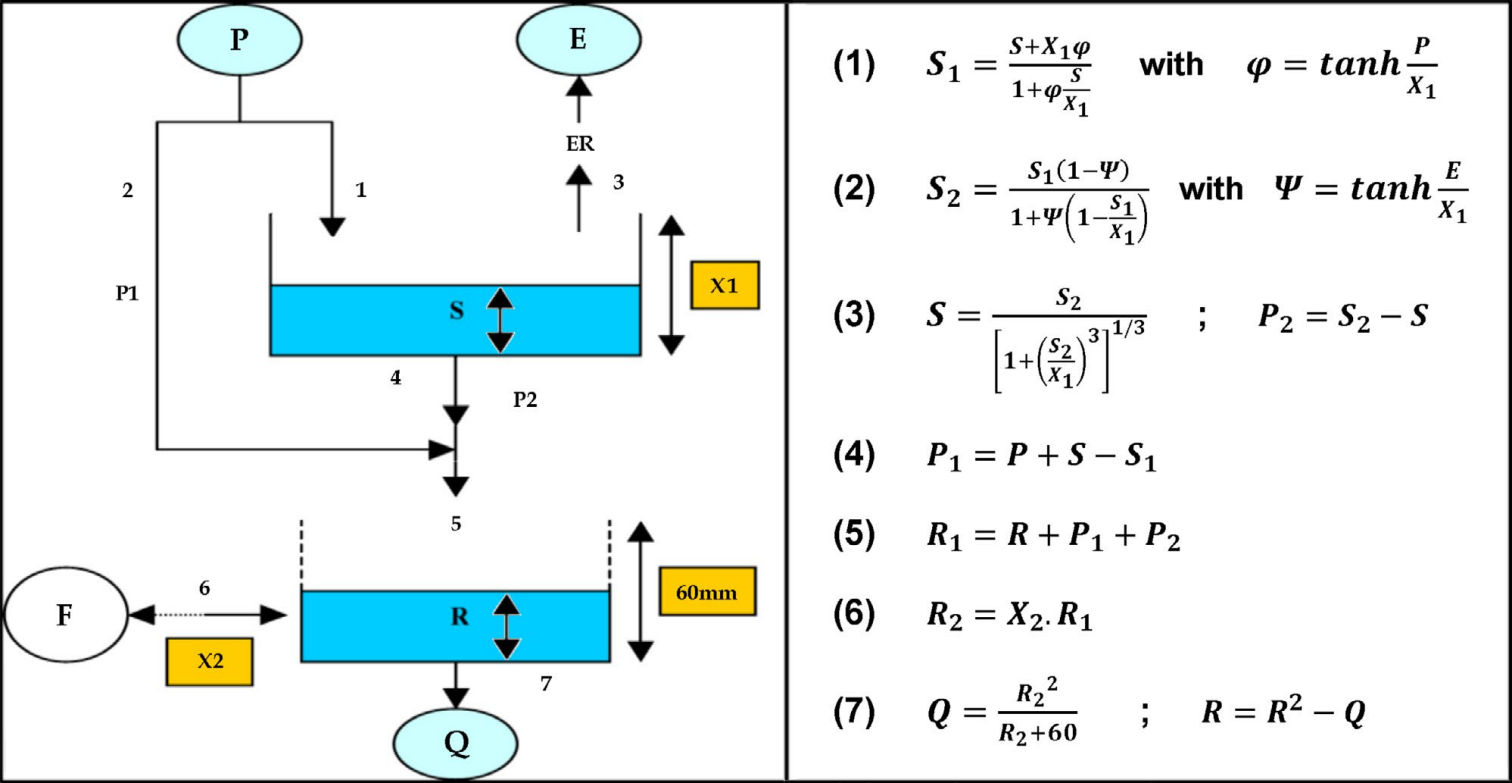
Outline of the structure of the GR2M model together with relevant equations defining the model structure. (Adapted from Mouelhi *et al*. (2013) and Lespinas *et al*. (2014)

GR2M is a simple water balance model (Mouelhi *et al*., 2006), originally developed for French catchments, now available via the airGR R hydrological modelling package (Coron *et al*., 2017). The monthly flow model contains two reservoirs representing a soil store and routing reservoir (Figure 4) governed by two parameters: the production store capacity and groundwater exchange coefficient. GR2M has been widely deployed across diverse catchment types and applications (e.g. Louvet *et al*., 2016), including for flow reconstructions (Dieppois *et al*., 2016).

For each catchment, GR2M was calibrated and validated on observed data before using the bias corrected Casty data to reconstruct flows. A split record for calibration/validation was applied as this allows direct comparison between GR2M and ANN model outputs on a catchment‐by‐catchment basis. Calibration for all catchments (including a 1‐year warm‐up period) was undertaken from the start of the flow record up to December 2000. This time interval captures periods of large flow variability ranging from the drought rich 1970s to the flood rich 1980s. Validation was undertaken using the 15 years postcalibration (2001–2016) for all catchments (see Table 1).

Uncertainty in GR2M model parameters was sampled using Monte Carlo methods. For each parameter, 20,000 values were randomly drawn from a uniform distribution of [0–2500] for the production store capacity and [0–2] for the groundwater exchange coefficient. Each parameter set was used to simulate flows for the calibration period (yielding a 20,000‐member ensemble). The performance of parameter sets was evaluated using two objective functions to ensure robust performance across the flow regime: the Nash Sutcliffe Efficiency (NSE) (Nash and Sutcliffe, 1970) derived from log transformed flows (logNSE) and the modified Kling Gupta Efficiency (KGE) derived from raw flows (Gupta *et al*., 2009; Kling *et al*., 2012). Two steps were then undertaken to determine which parameter sets to retain. First, objective function scores were ranked by their performance, with the top 400 sets from each being retained. Second, retained simulations were evaluated by their absolute per cent bias (PBIAS) relative to observed flows, with the 200 best performing parameter sets for both logNSE and KGE retained. The median (henceforth GR2M median) and 95th percentile confidence intervals of GR2M simulated flows, retained from this process, were then determined.

#### 2.3.2 The ANN Model

**FIGURE 5:**
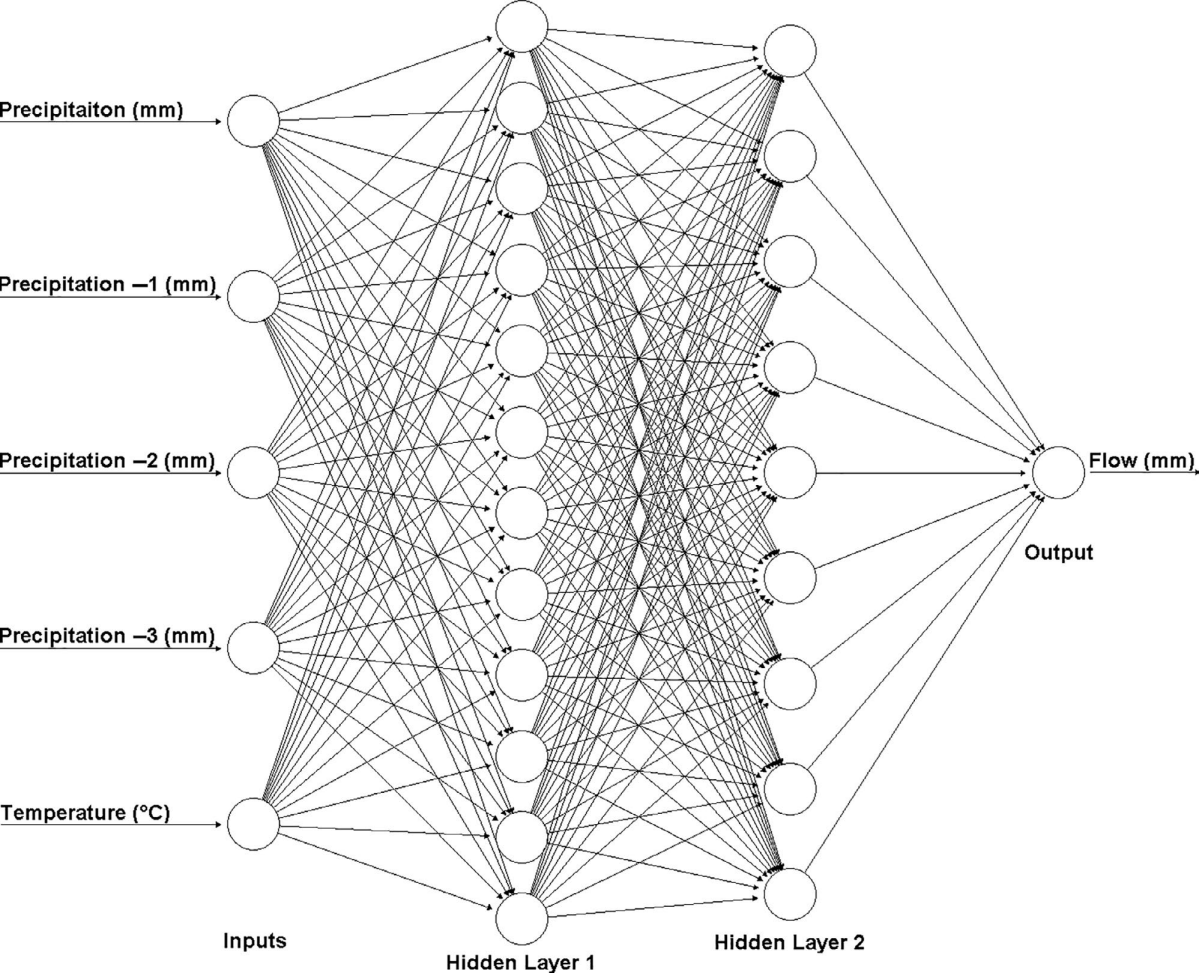
Schematic of a typical ANN model structure employed with five inputs, two hidden layers (with 12 and 9 neurons respectively) and monthly flow output. Negative one, two and three values represent the number of lagged months for precipitation

ANNs have been widely used for rainfall–runoff modelling (Dawson and Wilby, 1998; Dastorani *et al*., 2010). A backpropagation ANN was developed here using the neuralnet R package (Fritsch *et al*., 2019), with different combinations of inputs and neurons tested with two hidden layers. The same calibration and validation periods for individual catchments were employed as those for the GR2M, again using observed data to generate the model. When determining the ANN structure, input data were limited to observed variables that were also available for the full reconstruction period (temperature, precipitation and PET). Lagged variables (e.g. precipitation from previous months) were also included. The best performing ANN inputs were found to be temperature and precipitation from the current month, plus precipitation lagged by one, two and three months. An example ANN structure which generated the best efficiency scores for one catchment is shown in Figure 5.

Uncertainty in ANN model structure was explored by varying combinations of neurons in one or two hidden layers. Neuron permutations, varying from one to twenty for each hidden layer (giving 420 independent model structures in total), were used to simulate flows for the given calibration period. Each model structure was then independently evaluated using logNSE and KGE and ranked in order of performance. As per the GR2M model, the top 400 ANN model structures according to each objective function were identified and those which subsequently produced the 200 lowest PBIAS scores were retained. The median (henceforth ANN median) and 95th percentile confidence intervals of simulated flows were then obtained.

Finally, a mixed ensemble was derived from both GR2M and ANN model structures and parameters by combining the 200 retained simulations from each. The median (henceforth Ensemble median) and 95th percentile confidence intervals of simulated flows were obtained and used to evaluate model reconstructions.

#### 2.3.3 Validation results

**FIGURE 6:**
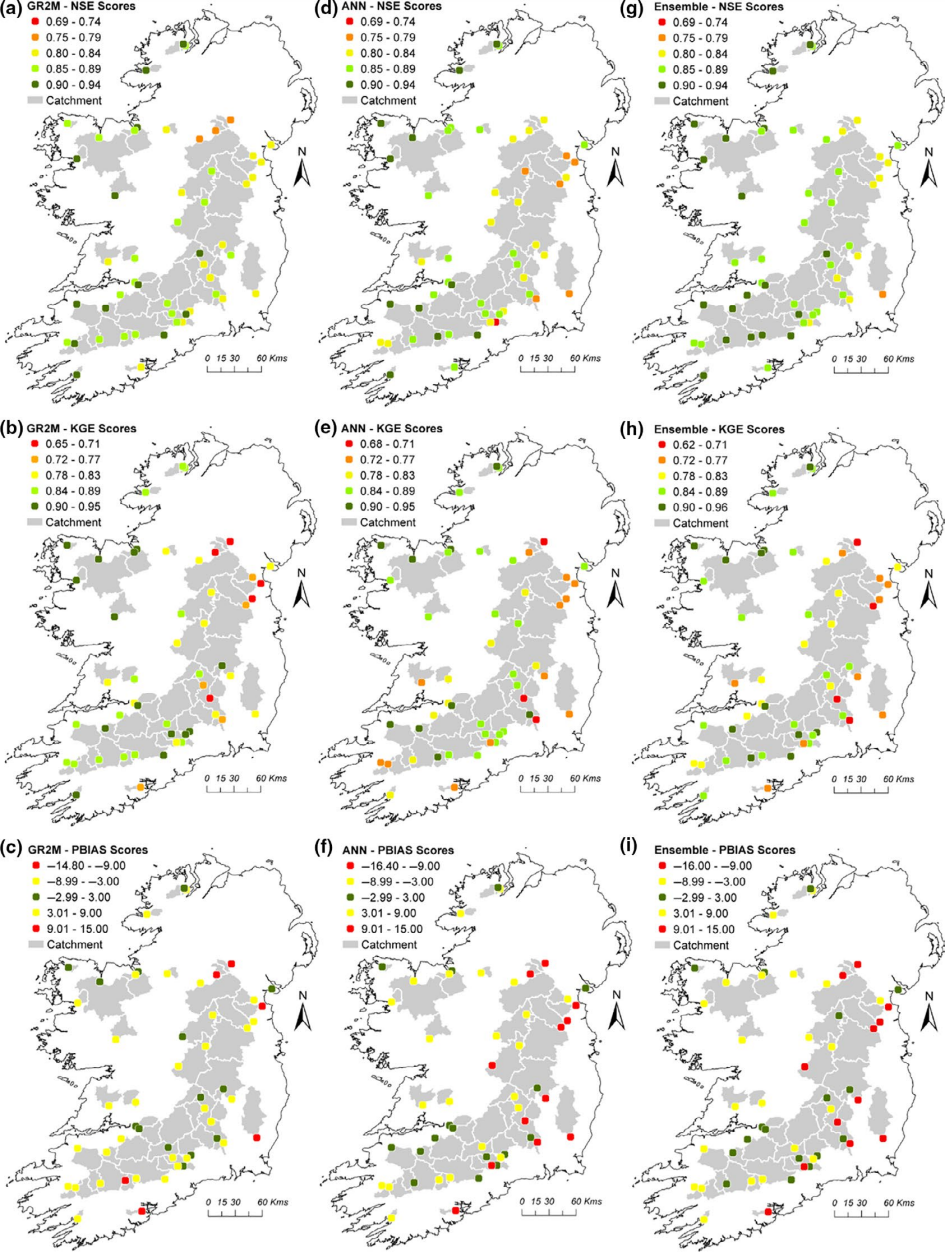
Maps of logNSE, KGE and PBIAS scores for GR2M, ANN and Ensemble median simulations for all 51 catchments. Scores are derived from the observed versus modelled flows for the independent validation period (2001–2016) for each catchment

Figure 6 displays the performance of the GR2M, ANN and Ensemble median simulations for all 51 catchments for the 2001–2016 validation period according to logNSE, KGE and PBIAS scores. The ANN and GR2M simulations perform equally well with average logNSE, KGE and PBIAS scores across all 51 catchments of 0.86, 0.83 and −3.04% for GR2M median and 0.85, 0.83 and −4.97% for ANN median. The combined Ensemble median returned scores of 0.87, 0.83 and −4.38%. Individual catchment results also show similar performance for both model types.

Skill scores for GR2M, ANN and Ensemble median simulations during validation for each catchment are provided in Table 1. Poorest performances are evident for the Nire at Fourmilewater (ID: 16013) which has a logNSE score of 0.69 (ANN median) and the Finn at Anlore (ID: 36015) with a KGE score of 0.65 (GR2M median). PBIAS scores vary between catchments with the largest bias evident for the Blackwater at Faulkland (ID: 3051) (−16.4%; ANN median) and a minimum of 0% for the Glenamoy at Glenamoy (ID: 33001) (GR2M median). PBIAS values are generally higher for the ANN median.

**FIGURE 7:**
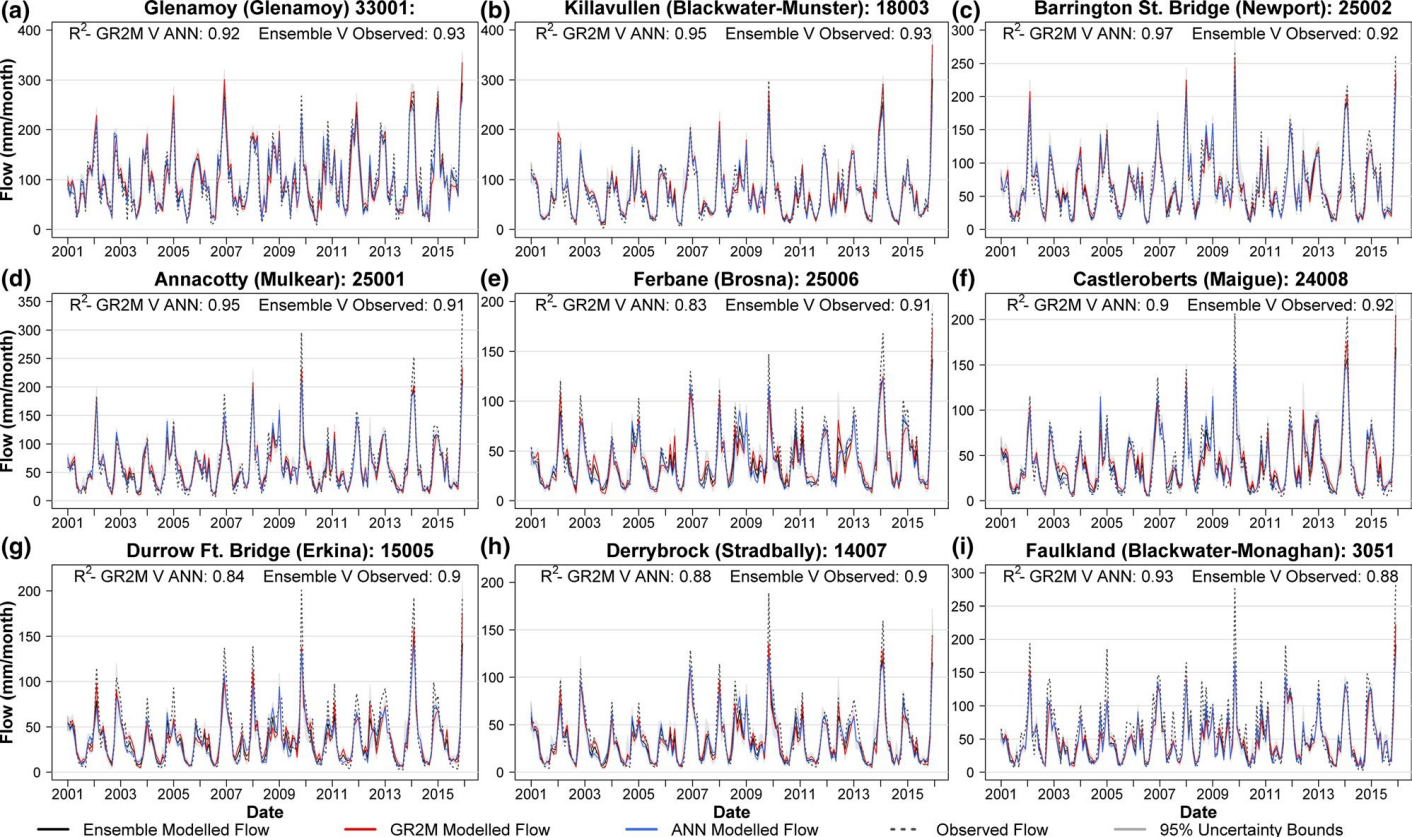
Observed and simulated annual mean flows for nine sample catchments representing best (top row), average (middle row) and worst (bottom row) performing models. Plotted are the GR2M (red), ANN (blue) and Ensemble median (black) simulations, together with observed flows (dashed dark‐grey). 95% uncertainty range (grey) is derived from the Ensemble median simulations

Observed and simulated monthly flows for the validation period for nine catchments are shown in Figure 7. This sub‐set represents a spread of the best (top row), average (middle row) and worst (bottom row) performing catchments. The proportion of observed variance (*R* ^2^) captured by the Ensemble median simulation for each catchment is also provided—varying between 0.88 and 0.93 for the nine sample catchments. The average Ensemble median *R* ^2^ value across all 51 catchments for the same validation period is 0.90. ANN and GR2M median simulations show good agreement for the majority of catchments. Whilst observed flows are largely contained within the uncertainty bounds for each of the catchment reconstructions, some discrepancies are apparent in peak values. Arterial drainage works have been identified as a probable cause of this, with previous work showing the tendency for elevated peak flows following drainage (Harrigan *et al*., 2014). Peak flows also tend to be underestimated for smaller catchments where gridded rainfall may not capture flood generating precipitation adequately.

## 3 RECONSTRUCTED FLOWS

### 3.1 Assessment of reconstructed flows

Following calibration and validation with observed data, bias corrected Casty data (precipitation/temperature and Oudin PET) were input to the hydrological models to reconstruct monthly river flows back to 1766. The following sub‐sections present the resulting annual, seasonal and monthly flow reconstructions across all 51 catchments.

#### 3.1.1 Annual flow reconstructions

**FIGURE 8:**
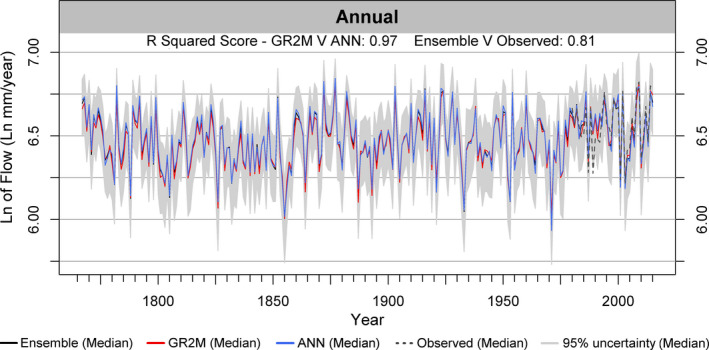
Median annual flow values across all 51 catchments for the period 1766–2016 for GR2M (red), ANN (blue) and Ensemble median (black) reconstructions. The median of observed flows across the catchment sample for years 1980–2016 are in dark‐grey, whilst 95% uncertainty ranges (grey) are derived from the ensemble simulations

The median of annual reconstructed flows for all 51 catchments from 1766 is shown in Figure 8. GR2M and ANN median reconstructions show close agreement (*R* ^2^ = 0.97). In Figure 8 and subsequent plots, observed flows from 1980 onward are displayed as, by this year, observed values are available for over 84% of catchments. Overall, the percentage of median annual observed flow values across all 51 catchments contained within the uncertainty ranges of the median ensemble (henceforth the containment value) is 97%. Observed and Ensemble median simulated series across all catchments show close agreement (*R* ^2^ = 0.81). Some divergence is evident between modelled and observed flows around 1989 due to differences between Casty and observed precipitation at that time.

#### 3.1.2 Seasonal and monthly flow reconstructions

**FIGURE 9:**
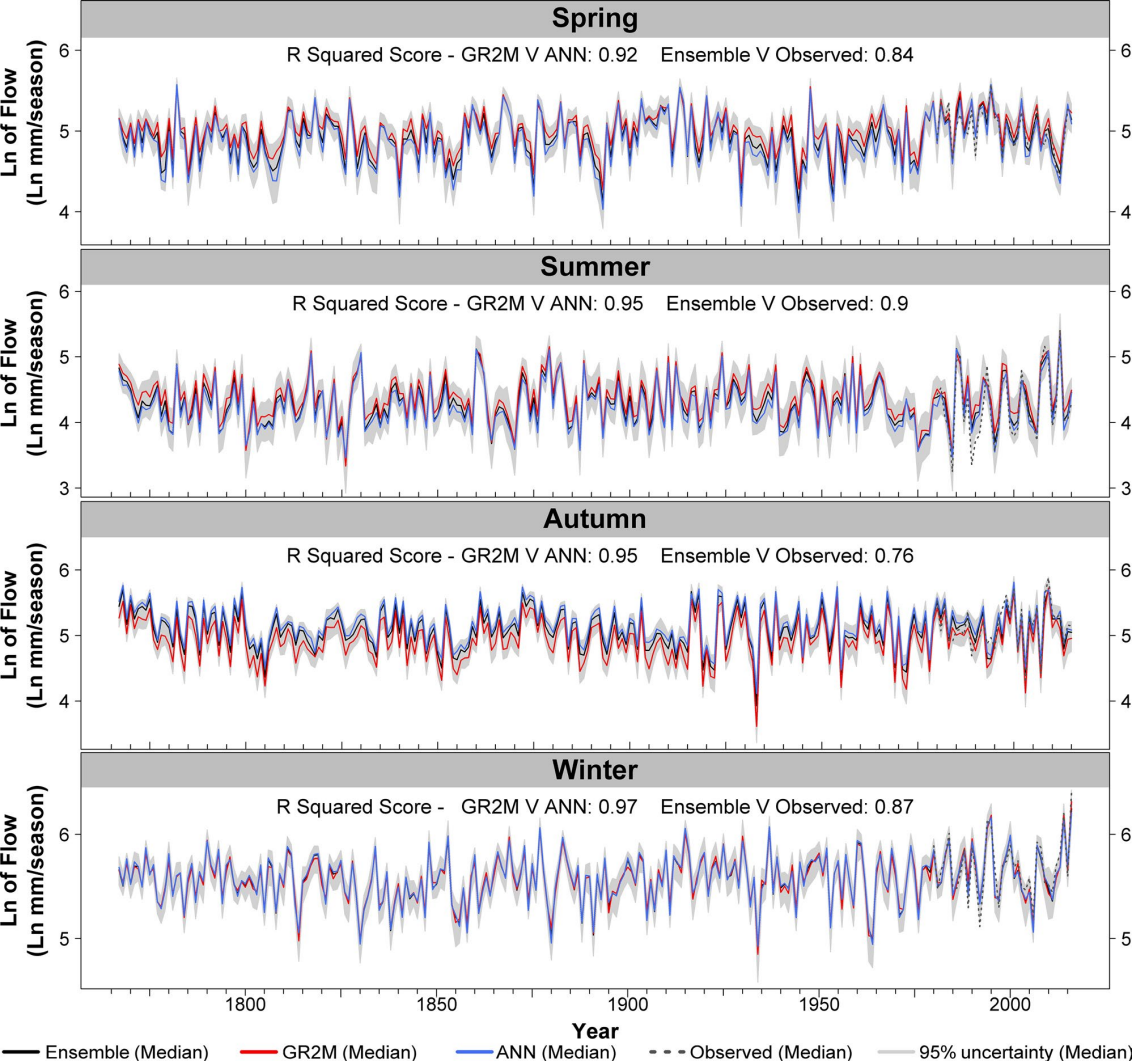
As in Figure 8 but for seasonal median flows: Winter [DJF], spring [MAM], summer [JJA], autumn [SON]

**FIGURE 10:**
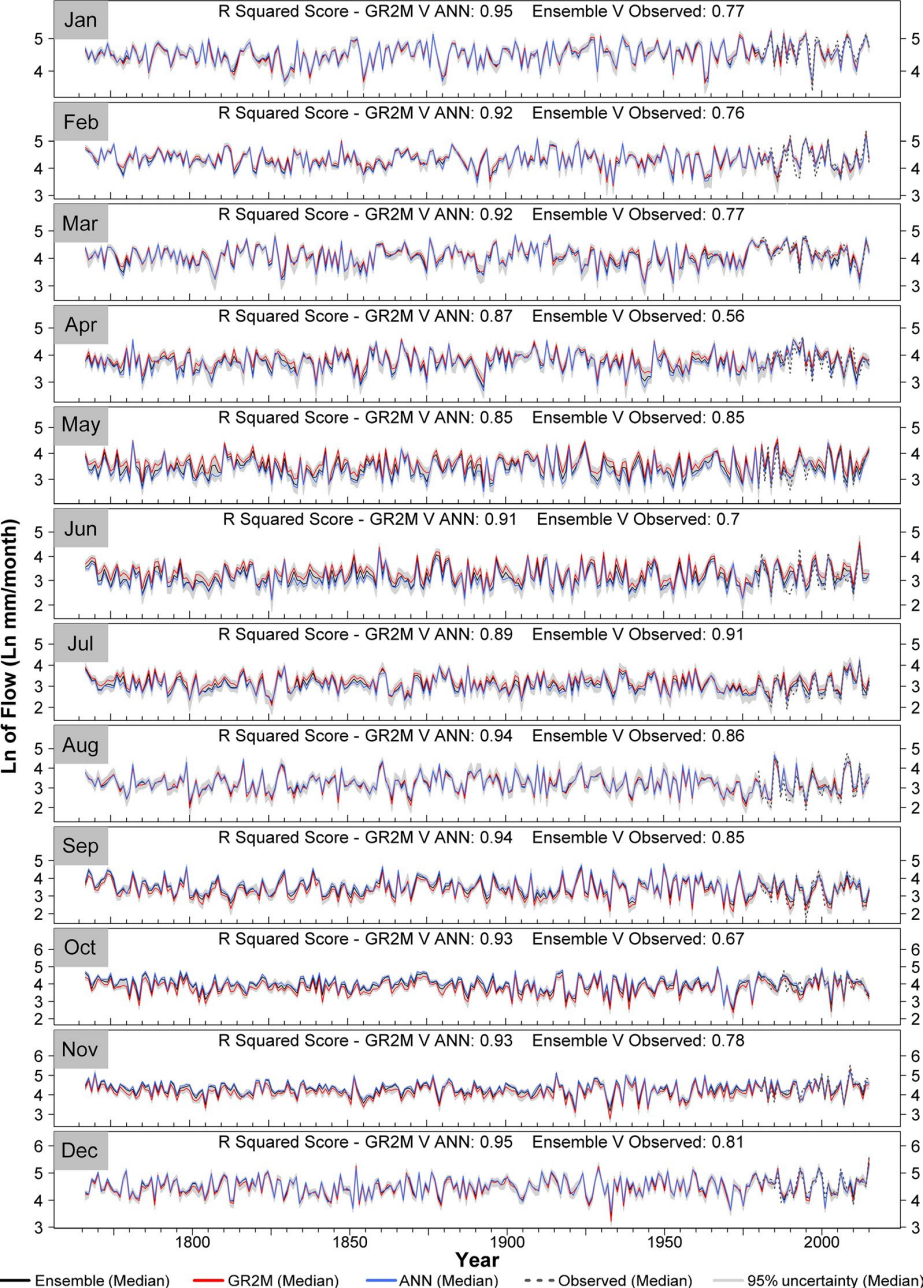
As in Figure 8 but for monthly median flows

Seasonal and monthly flow reconstructions for all 51 catchments are displayed in Figures 9 and 10, respectively, with reconstructions showing strong agreement with observations for 1980–2016 in all seasons. There is some evidence that summer flows are over‐estimated in 1989, consistent with annual flows. For all other periods and seasons, observed flows lie within uncertainty estimates (minimum containment value is 89%) and show good agreement with reconstructions (*R* ^2^ between Ensemble median values and observations range from a high of 0.9 in summer [JJA] to a low of 0.76 in autumn [SON]). Close agreement is also evident between GR2M and ANN median reconstructions (*R* ^2^ > 0.91) in all seasons. It is notable from Figure 9 that GR2M reconstructions for spring and summer are slightly higher and autumn values lower than ANN reconstructions.

Monthly reconstructions are displayed in Figure 10 for all 51 catchments. Good agreement is evident between GR2M and ANN median reconstructions (*R* ^2^ > 0.84 in all months). GR2M median reconstructions are slightly higher than the ANN in April, May, June and July, whilst GR2M output in September, October and November is lower than the ANN equivalent, concurrent with summer and autumn differences between GR2M and ANN values identified above. As expected, performance of monthly simulations is poorer than for seasonal and annual time steps. Monthly observed flows generally lie within uncertainty estimates (mean containment value across all months is 68%) and show satisfactory agreement with observations (*R* ^2^ for Ensemble median values vs. observations range between 0.56 in April and 0.91 in July).

### 3.2 Comparison with reconstructions from long‐term precipitation series

Monthly river flow reconstructions generated with the bias corrected Casty data were evaluated against reconstructions based on monthly precipitation data for stations within the Island of Ireland Precipitation (IIP) network 1850–2010 (Noone *et al*., 2016). For each catchment, we identified the nearest IIP station (see Figure 1) and then bias corrected data to catchment average precipitation, as per the Casty data. Bias corrected precipitation, together with bias corrected monthly temperature/PET derived from the Casty data, was used to reconstruct flows back to 1850, using the same methods as described above. Although some of the IIP data are likely contained within the Casty gridded precipitation (so there is a degree of circularity), it was deemed important to compare both data sources, given the different methods used in their construction.

**FIGURE 11:**
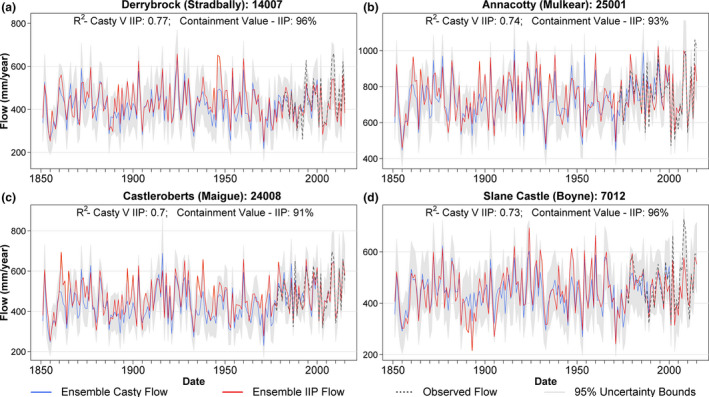
Reconstructed annual mean flow values for four sample catchments. Ensemble median simulations generated using Casty precipitation data (blue), and Island of Ireland Precipitation (IIP) data (red), together with observed flows (dashed dark‐grey) are displayed for each catchment

Figure 11 shows the Ensemble median annual mean flow reconstructions from 1850 to 2016 for four exemplar catchments, using Casty precipitation or IIP as input. Strong agreement between the reconstructions is evident despite the different input data with IIP reconstructions largely contained within the uncertainty ranges of the Casty reconstructions. Across the four case study catchments, the *R* ^2^ between IIP and Casty reconstructed annual mean flows varies between 0.70 and 0.77. Differences between flows generated from the two data sources are not unexpected given that IIP data are station based and often located outside catchment boundaries, whereas Casty data are gridded.

### 3.3 High‐ and low‐flow assessment

The most notable extreme flow years for seasonal and annual Casty reconstructions were identified (Table 2), with the top five highest and lowest flow year across all catchments displayed for calendar years (1767–2016) as well as winter and summer seasons (1767–2016). The percentage anomaly relative to the mean of the full record is also provided. The most exceptional high‐flow years across the sample include 1877, 1872 and 1916, whilst the most notable winter seasons include 2015/16, 1994/95 and 2013/14. In terms of exceptional low‐flow years, 1855, 1933 and 1971 standout across the catchments, whereas 1826, 1975 and 1887 dominate the most notable low‐flow years for summer. Annual flow anomalies across all 51 catchments range from 150% to 58% of the long‐term mean for all catchments, whilst seasonally winter and summer extreme anomalies range from 173% to 37% of the respective long‐term seasonal mean values. Our extreme years and seasons show considerable agreement with a similar evaluation of reconstructed river flows (1865–2002) in the United Kingdom (Jones *et al*., 2006), with the previously identified exceptional high‐ and low‐flow seasons and years (1865–2002) all found at least once in the top five equivalent events for multiple catchments in that series.

**Table 2:** Years with the five highest and lowest annual (calendar), winter [DJF] (year given for January) and summer [JJA] flows for the period of reconstructions 1767–2016 across all 51 catchments

**Station (ID)**	**Top 5 High Flow Years**										**Top 5 Low Flow Years**									
	**Annual**					**Winter (December to February)**					**Annual**					**Summer (June to August)**				
	1	2	3	4	5	1	2	3	4	5	1	2	3	4	5	1	2	3	4	5
**3051**	**2015**	**2002**	**1877**	**1954**	**2009**	**2016**	**1995**	**1994**	**2014**	**1937**	**1933**	**1826**	**1855**	**1953**	**1971**	**1826**	**1975**	**1983**	**1800**	**1870**
	150%	145%	144%	138%	137%	193%	172%	166%	161%	159%	59%	62%	62%	65%	65%	33%	33%	35%	37%	39%
**6013**	**1877**	**1966**	**2002**	**1782**	**1872**	**2016**	**1877**	**1994**	**1883**	**1915**	**1953**	**1971**	**1933**	**1975**	**1855**	**1975**	**1826**	**1887**	**1995**	**1870**
	154%	150%	146%	141%	138%	170%	158%	154%	151%	151%	53%	57%	62%	63%	64%	30%	32%	38%	39%	42%
**6014**	**2002**	**1877**	**1966**	**1782**	**1872**	**2016**	**1877**	**1883**	**1994**	**1915**	**1953**	**1971**	**1855**	**1933**	**1826**	**1975**	**1826**	**1995**	**1887**	**1870**
	160%	152%	146%	138%	136%	174%	153%	151%	151%	148%	55%	57%	63%	63%	64%	35%	36%	43%	44%	47%
**6030**	**2002**	**1877**	**2015**	**1782**	**1872**	**2016**	**2013**	**1994**	**1877**	**1990**	**1933**	**1826**	**1953**	**1887**	**1911**	**1995**	**1800**	**1826**	**1949**	**1983**
	173%	142%	141%	137%	137%	201%	173%	163%	161%	158%	61%	64%	65%	67%	69%	24%	28%	28%	31%	31%
**7009**	**1877**	**1924**	**1782**	**1872**	**1960**	**2016**	**2014**	**1877**	**1995**	**1994**	**1971**	**1953**	**1855**	**1933**	**1826**	**1975**	**1826**	**1995**	**1887**	**1870**
	142%	137%	135%	135%	133%	173%	151%	147%	146%	145%	57%	61%	66%	67%	68%	36%	38%	42%	44%	45%
**7012**	**1877**	**1924**	**1782**	**1872**	**1965**	**2016**	**1877**	**2014**	**1937**	**1995**	**1971**	**1953**	**1855**	**1933**	**1826**	**1975**	**1826**	**1887**	**1995**	**1870**
	152%	141%	140%	138%	138%	180%	164%	163%	161%	158%	56%	60%	64%	64%	65%	32%	34%	41%	41%	43%
**12001**	**1960**	**1930**	**1872**	**1924**	**2009**	**2016**	**1877**	**1995**	**1994**	**1930**	**1971**	**1953**	**1905**	**1788**	**1855**	**1975**	**1826**	**1995**	**1887**	**1984**
	158%	150%	148%	146%	145%	192%	177%	177%	170%	169%	55%	62%	64%	66%	66%	37%	38%	42%	44%	44%
**14007**	**1924**	**1877**	**1872**	**1960**	**1903**	**1915**	**2014**	**1995**	**2016**	**1883**	**1971**	**1953**	**1855**	**1788**	**1933**	**1975**	**1826**	**1995**	**1887**	**1870**
	155%	145%	144%	143%	141%	171%	169%	168%	165%	160%	53%	62%	64%	66%	67%	38%	40%	43%	45%	47%
**14019**	**1924**	**1960**	**2008**	**1877**	**1872**	**2014**	**2016**	**1995**	**1915**	**1994**	**1971**	**1953**	**1855**	**1905**	**1933**	**1975**	**1826**	**1995**	**1887**	**1870**
	149%	144%	142%	141%	140%	175%	173%	170%	165%	162%	57%	65%	67%	69%	70%	43%	46%	47%	52%	53%
**15001**	**1877**	**1872**	**1924**	**1928**	**1903**	**1930**	**1995**	**1877**	**1915**	**1937**	**1971**	**1953**	**1855**	**1788**	**1826**	**1826**	**1975**	**1984**	**1870**	**1887**
	160%	155%	153%	152%	151%	193%	192%	190%	190%	184%	51%	54%	59%	60%	64%	32%	33%	35%	36%	36%
**15003**	**1924**	**2009**	**1872**	**1877**	**1916**	**2014**	**1995**	**1915**	**2016**	**1937**	**1971**	**1953**	**1788**	**1855**	**1933**	**1826**	**1975**	**1870**	**1887**	**1995**
	150%	148%	147%	147%	147%	179%	177%	173%	164%	162%	50%	57%	61%	64%	64%	24%	24%	27%	28%	28%
**15005**	**1877**	**1872**	**1903**	**1916**	**1924**	**1930**	**1915**	**1937**	**2016**	**1877**	**1953**	**1971**	**1855**	**1788**	**1933**	**1826**	**1984**	**1887**	**1975**	**1870**
	152%	145%	142%	142%	141%	180%	175%	174%	174%	171%	57%	61%	62%	67%	67%	39%	40%	43%	43%	46%
**15006**	**1877**	**1872**	**1924**	**1928**	**1903**	**1915**	**1995**	**2016**	**1930**	**1877**	**1971**	**1953**	**1855**	**1788**	**1933**	**1826**	**1975**	**1984**	**1887**	**1870**
	152%	146%	145%	144%	143%	178%	178%	176%	174%	172%	57%	60%	65%	66%	68%	39%	41%	42%	44%	45%
**15007**	**1872**	**1877**	**1954**	**1903**	**1924**	**2016**	**1995**	**1937**	**2014**	**1915**	**1971**	**1953**	**1855**	**1933**	**1826**	**1826**	**1984**	**1800**	**1870**	**1975**
	142%	142%	141%	140%	140%	177%	173%	172%	172%	168%	57%	58%	59%	61%	65%	37%	39%	43%	43%	44%
**16008**	**1877**	**1872**	**1903**	**1923**	**1954**	**2016**	**1937**	**1995**	**1877**	**2014**	**1953**	**1855**	**1971**	**1933**	**1826**	**1984**	**1826**	**1870**	**1887**	**1975**
	144%	143%	140%	139%	139%	180%	168%	167%	161%	161%	58%	59%	60%	65%	67%	38%	40%	44%	47%	48%
**16009**	**1877**	**2009**	**1872**	**1903**	**1916**	**2016**	**1995**	**1937**	**1915**	**2014**	**1855**	**1971**	**1953**	**1933**	**1826**	**1826**	**1984**	**1870**	**1975**	**1887**
	147%	144%	143%	143%	142%	189%	184%	175%	172%	170%	60%	61%	62%	66%	68%	45%	45%	51%	51%	52%
**16010**	**2009**	**1877**	**1928**	**1903**	**1916**	**1930**	**2016**	**1915**	**1995**	**1994**	**1855**	**1971**	**1788**	**1953**	**1893**	**1826**	**1984**	**1870**	**1975**	**1887**
	153%	152%	152%	148%	145%	189%	189%	187%	181%	179%	61%	64%	66%	67%	68%	44%	47%	50%	50%	51%
**16011**	**1877**	**1903**	**2009**	**1928**	**1872**	**2016**	**1995**	**1915**	**1994**	**1937**	**1971**	**1855**	**1953**	**1933**	**1788**	**1826**	**1984**	**1975**	**1870**	**1887**
	143%	141%	141%	140%	139%	185%	181%	172%	168%	167%	64%	65%	67%	69%	71%	51%	52%	56%	57%	57%
**16012**	**1928**	**2009**	**1877**	**1903**	**1960**	**1995**	**2016**	**1915**	**1930**	**1925**	**1855**	**1971**	**1826**	**1933**	**1887**	**1826**	**1984**	**1887**	**1975**	**1870**
	143%	141%	140%	138%	138%	188%	187%	170%	169%	168%	65%	66%	70%	71%	72%	47%	50%	53%	53%	56%
**16013**	**1928**	**2009**	**1938**	**1903**	**1960**	**1930**	**1995**	**2016**	**1915**	**1994**	**1971**	**1933**	**1855**	**1788**	**1887**	**1826**	**1984**	**1870**	**1800**	**1887**
	152%	148%	147%	145%	145%	196%	185%	185%	184%	182%	63%	66%	67%	68%	69%	38%	40%	43%	45%	46%
**18002**	**1916**	**2009**	**1877**	**2000**	**1872**	**2016**	**2014**	**1995**	**1915**	**1994**	**1971**	**1855**	**1933**	**1788**	**1826**	**1826**	**1984**	**1887**	**1975**	**1870**
	151%	142%	140%	139%	135%	209%	188%	184%	163%	162%	56%	61%	69%	70%	71%	44%	45%	50%	50%	51%
**18003**	**1916**	**2009**	**2000**	**1877**	**1994**	**2016**	**2014**	**1995**	**1915**	**1994**	**1971**	**1855**	**1788**	**1826**	**1921**	**1984**	**1826**	**1887**	**1975**	**1870**
	150%	143%	141%	138%	138%	215%	199%	189%	170%	167%	54%	61%	67%	69%	69%	38%	39%	45%	45%	47%
**18006**	**1916**	**2009**	**2000**	**1994**	**2002**	**2016**	**2014**	**1995**	**1915**	**1994**	**1971**	**1855**	**1826**	**1933**	**1788**	**1984**	**1826**	**1887**	**1975**	**1800**
	145%	144%	141%	140%	139%	217%	205%	191%	177%	173%	58%	62%	68%	69%	70%	42%	43%	47%	47%	48%
**18050**	**2009**	**2002**	**2008**	**2000**	**1872**	**2016**	**2014**	**1995**	**1915**	**1994**	**1971**	**1855**	**1788**	**1826**	**1921**	**1826**	**1984**	**1800**	**1975**	**1944**
	142%	139%	139%	138%	137%	203%	200%	185%	179%	172%	58%	62%	69%	69%	70%	40%	41%	42%	43%	44%
**19001**	**2009**	**1982**	**1928**	**2000**	**1872**	**2016**	**1995**	**2014**	**1915**	**1994**	**1971**	**1855**	**1788**	**1933**	**1854**	**1984**	**1826**	**1887**	**1870**	**1975**
	159%	153%	151%	150%	149%	233%	211%	203%	188%	187%	48%	57%	63%	63%	65%	37%	38%	40%	41%	42%
**21002**	**2009**	**1982**	**1872**	**1914**	**1916**	**2016**	**1995**	**1915**	**2014**	**1994**	**1971**	**1855**	**1955**	**1788**	**1887**	**1800**	**1976**	**1955**	**1975**	**1864**
	142%	136%	134%	134%	134%	203%	178%	168%	167%	164%	60%	70%	71%	72%	72%	33%	39%	42%	42%	46%
**22006**	**1916**	**1872**	**1982**	**2000**	**2002**	**2016**	**2014**	**1995**	**1915**	**1994**	**1971**	**1855**	**1933**	**1788**	**1921**	**1800**	**1976**	**1975**	**1984**	**2006**
	137%	135%	135%	135%	135%	199%	187%	183%	175%	169%	62%	66%	69%	71%	72%	45%	47%	51%	54%	54%
**22035**	**2000**	**1872**	**1916**	**1982**	**1994**	**2016**	**2014**	**1995**	**1915**	**1994**	**1971**	**1855**	**1933**	**1788**	**1921**	**1800**	**1975**	**1984**	**1826**	**1976**
	137%	136%	135%	135%	131%	202%	182%	176%	168%	163%	62%	67%	72%	73%	73%	51%	53%	56%	57%	58%
**23002**	**2008**	**2015**	**1986**	**1916**	**1872**	**2014**	**2016**	**1995**	**1915**	**1994**	**1971**	**1855**	**1788**	**1826**	**1921**	**1800**	**1984**	**1826**	**1975**	**1976**
	157%	145%	141%	140%	138%	215%	205%	195%	187%	177%	56%	61%	68%	68%	68%	35%	38%	39%	39%	39%
**24008**	**1916**	**2014**	**2009**	**1986**	**2008**	**2016**	**2014**	**1995**	**1994**	**1915**	**1971**	**1855**	**1933**	**1788**	**1826**	**1984**	**1826**	**1975**	**1887**	**1870**
	160%	154%	151%	148%	148%	228%	224%	212%	180%	176%	53%	58%	63%	66%	66%	37%	38%	42%	43%	45%
**24030**	**1916**	**1986**	**1994**	**2000**	**2008**	**2014**	**2016**	**1995**	**1994**	**1915**	**1971**	**1855**	**1826**	**1933**	**1921**	**1984**	**1826**	**1887**	**1870**	**1975**
	156%	153%	151%	151%	150%	228%	226%	213%	189%	188%	51%	56%	64%	64%	65%	35%	36%	42%	44%	44%
**25001**	**1916**	**2008**	**1986**	**1954**	**1877**	**1995**	**2016**	**2014**	**1937**	**1994**	**1855**	**1933**	**1971**	**1953**	**1826**	**1984**	**1826**	**1800**	**1870**	**2006**
	141%	140%	138%	137%	136%	185%	180%	177%	163%	161%	63%	63%	63%	67%	69%	42%	44%	47%	48%	50%
**25002**	**2008**	**1872**	**1877**	**1994**	**1916**	**1995**	**2016**	**2014**	**1994**	**1937**	**1971**	**1933**	**1855**	**1953**	**1826**	**1984**	**1826**	**1800**	**1870**	**2006**
	141%	138%	137%	135%	134%	186%	167%	164%	163%	162%	62%	64%	65%	67%	68%	30%	34%	38%	39%	41%
**25006**	**2014**	**1877**	**1872**	**1924**	**1954**	**2016**	**2014**	**1937**	**1995**	**1877**	**1971**	**1855**	**1933**	**1953**	**1826**	**1826**	**1975**	**1984**	**1995**	**1887**
	144%	143%	140%	140%	138%	194%	181%	179%	168%	163%	59%	66%	66%	67%	68%	44%	46%	47%	50%	51%
**25030**	**2015**	**1986**	**2014**	**2009**	**1994**	**2016**	**2014**	**1995**	**1994**	**1937**	**1855**	**1933**	**1971**	**2003**	**1826**	**1984**	**1826**	**1800**	**2006**	**1870**
	157%	145%	144%	140%	139%	219%	210%	201%	177%	162%	59%	63%	63%	63%	66%	39%	40%	41%	42%	46%
**25034**	**2014**	**1872**	**1924**	**1960**	**1877**	**2016**	**2014**	**1995**	**1915**	**1937**	**1971**	**1855**	**2003**	**1826**	**1933**	**1975**	**1826**	**1995**	**1887**	**1870**
	145%	140%	140%	140%	139%	190%	165%	158%	154%	152%	54%	66%	67%	68%	68%	30%	33%	35%	40%	41%
**26021**	**2015**	**1924**	**2014**	**1877**	**2002**	**2016**	**2014**	**1937**	**2007**	**1995**	**1971**	**1855**	**1933**	**1826**	**1805**	**1975**	**1826**	**1984**	**1887**	**1995**
	141%	139%	139%	138%	135%	196%	163%	159%	153%	148%	58%	65%	65%	67%	70%	35%	37%	42%	44%	45%
**26029**	**2015**	**1986**	**2002**	**1992**	**1877**	**2016**	**2014**	**1995**	**1937**	**1994**	**1933**	**1855**	**2001**	**1826**	**1805**	**1800**	**1826**	**1984**	**1821**	**1983**
	144%	138%	134%	133%	131%	183%	173%	164%	154%	152%	64%	65%	65%	69%	73%	30%	34%	34%	35%	37%
**26058**	**2002**	**1877**	**1924**	**2015**	**2014**	**2016**	**2014**	**1937**	**2007**	**1995**	**1971**	**1855**	**1933**	**1826**	**1805**	**1975**	**1826**	**1887**	**1995**	**1984**
	143%	140%	139%	138%	136%	195%	166%	153%	144%	143%	58%	62%	62%	65%	68%	33%	34%	42%	43%	44%
**27002**	**2009**	**2015**	**2008**	**1872**	**1986**	**2016**	**2014**	**1995**	**1994**	**1915**	**1855**	**1971**	**1933**	**1921**	**1826**	**1984**	**1826**	**1975**	**1800**	**1887**
	160%	150%	148%	139%	139%	221%	199%	192%	168%	165%	57%	58%	63%	64%	66%	33%	34%	39%	40%	41%
**30007**	**2015**	**1986**	**1950**	**1954**	**2002**	**2016**	**2014**	**1995**	**1937**	**1994**	**1855**	**1933**	**1826**	**1805**	**1921**	**1826**	**1984**	**1887**	**1870**	**1978**
	154%	148%	140%	140%	138%	197%	169%	165%	164%	153%	58%	62%	65%	68%	68%	38%	39%	46%	49%	49%
**32012**	**2015**	**1986**	**2008**	**1949**	**2011**	**2016**	**2014**	**1995**	**1994**	**1937**	**1855**	**1933**	**1826**	**1805**	**1971**	**1800**	**1826**	**1984**	**1983**	**1976**
	147%	142%	137%	130%	130%	180%	178%	165%	154%	151%	64%	65%	71%	73%	73%	42%	46%	48%	50%	52%
**33001**	**2015**	**1986**	**2008**	**1992**	**1949**	**2014**	**2016**	**1995**	**1994**	**2000**	**1855**	**1933**	**1826**	**1805**	**1971**	**1800**	**1983**	**1995**	**1984**	**1968**
	149%	141%	131%	128%	127%	185%	177%	166%	151%	147%	65%	66%	74%	76%	76%	34%	35%	44%	45%	46%
**34001**	**2015**	**1986**	**1950**	**2008**	**1877**	**2016**	**2014**	**1995**	**1937**	**1950**	**1855**	**1933**	**1805**	**1826**	**2003**	**1826**	**1984**	**1975**	**1800**	**1887**
	143%	141%	138%	138%	136%	185%	175%	159%	156%	149%	60%	63%	68%	69%	70%	41%	45%	49%	51%	51%
**35002**	**2015**	**2008**	**2002**	**1986**	**1949**	**2016**	**2014**	**1995**	**2015**	**1994**	**1855**	**1933**	**1805**	**1826**	**1921**	**1800**	**1826**	**1995**	**1983**	**1984**
	150%	146%	145%	136%	132%	187%	181%	167%	156%	154%	63%	64%	70%	70%	75%	40%	43%	43%	47%	47%
**35005**	**2015**	**2002**	**2008**	**1986**	**1950**	**2016**	**2014**	**1937**	**1995**	**2015**	**1855**	**1933**	**1805**	**1826**	**1921**	**1826**	**1984**	**1800**	**1995**	**1975**
	149%	144%	143%	140%	137%	190%	186%	164%	163%	153%	62%	63%	66%	66%	69%	38%	42%	46%	46%	47%
**36015**	**1877**	**1954**	**1998**	**2002**	**1782**	**1995**	**2016**	**1937**	**1994**	**1877**	**1933**	**1826**	**1855**	**1971**	**1921**	**1975**	**1826**	**1983**	**1800**	**1870**
	146%	145%	142%	141%	138%	169%	164%	158%	157%	153%	57%	60%	60%	63%	65%	27%	28%	31%	32%	33%
**36019**	**2002**	**1877**	**2015**	**1954**	**1998**	**2016**	**2014**	**1937**	**1995**	**2007**	**1971**	**1933**	**1826**	**1855**	**1953**	**1826**	**1975**	**1984**	**1870**	**1887**
	155%	146%	141%	139%	138%	179%	166%	158%	154%	153%	58%	59%	61%	61%	65%	24%	24%	31%	33%	34%
**38001**	**2015**	**1949**	**2011**	**1992**	**1986**	**1995**	**2014**	**2016**	**1994**	**2015**	**1933**	**1855**	**1805**	**1826**	**1864**	**1800**	**1821**	**1995**	**1983**	**1984**
	145%	139%	136%	132%	131%	183%	180%	165%	162%	160%	63%	64%	69%	70%	75%	29%	37%	37%	40%	40%
**39006**	**2015**	**1990**	**1949**	**1992**	**1999**	**2016**	**1995**	**2015**	**2014**	**1994**	**1855**	**1933**	**1826**	**1805**	**1911**	**1800**	**1995**	**1983**	**1824**	**1984**
	167%	141%	138%	138%	137%	189%	185%	179%	176%	173%	60%	63%	66%	67%	70%	34%	34%	36%	37%	38%
**39009**	**2015**	**1949**	**1990**	**1992**	**1999**	**2016**	**1995**	**2015**	**2014**	**1994**	**1855**	**1933**	**1805**	**1826**	**1911**	**1800**	**1983**	**1995**	**1984**	**1824**
	165%	140%	140%	138%	138%	186%	184%	177%	175%	173%	60%	62%	67%	67%	71%	32%	34%	34%	35%	36%

Note

The percentage anomaly relative to the long‐term mean (1767–2016) is provided in each case. Values highlighted in progressively darker blue represent the top three occurring high flow events, whilst those in red represent the top three occurring low‐flow events.

## 4 DATA SET ACCESS, USES AND LIMITATIONS

The derived monthly flow reconstructions (December 1766 to November 2016 inclusive) for the 51 catchments are freely available for download from the PANGAEA data centre (https://doi.org/10.1594/PANGAEA.914306). Data are presented as five individual tab‐delimited text files (ASCII), representing reconstructions for each catchment from the GR2M, ANN and Ensemble median simulations, along with 2.5% and 97.5% quantiles derived from the Ensemble simulation. Also included is a table providing the geographical co‐ordinates of all 51 flow stations.

### 4.1 Potential uses

The reconstructed flow series provide a resource for assessing the impacts of extreme meteorological events, such as drought, on river flows across Ireland, extending the work of Noone *et al*. (2017) and Noone and Murphy (2020). Our reconstructions could also inform spatio‐temporal assessments of variability plus support detection of multi‐centennial changes in river flows (e.g. Wilby, 2006). Furthermore, the multi‐centennial time scale of our reconstructions offers the potential to examine how modes of ocean and climate variability influence river flows over extended periods. For example, it is known that Atlantic multidecadal variability exerts an important control on Ireland's climate (McCarthy *et al*., 2015), but its impact on river flows is less clear. Our long‐term data set offers the means to explore any potential control, including its stationarity. In turn, this could help facilitate improved seasonal forecasting (e.g. Wedgbrow *et al*., 2002).

This work represents the first reconstruction of monthly flows for a large number of Irish catchments using long‐term reanalysis data and observations. Given the uncertainties involved, this data set should be treated as a benchmark and evaluated and improved by future products. The approach to flow reconstruction adopted here is easily transferable to other catchments in Europe (i.e. the domain of Casty data). By taking advantage of observed runoff data, available from the Global Runoff Data Centre (https://www.bafg.de/GRDC/EN/Home/homepage_node.html), it would be possible to generate similar archives of monthly flow reconstructions for the entire continent.

### 4.2 Limitations

There are several recognized limitations to reconstructed river flows. First, arterial drainage has had a pervasive impact on Irish rivers. Catchments in this data set that have been drained tend to have higher peak flows during winter months than captured by the reconstructions. This is consistent with the findings of Harrigan *et al*. (2014) for the Boyne catchment. Hence, our reconstructions may be useful for quantifying the impact of arterial drainage on flow response. Moreover, we note that there is limited knowledge about how arterial drainage affects low‐flow and drought responses—again, our reconstructions may provide a useful point of reference.

Changes in land use can have considerable impacts on flows over time (Yan *et al*., 2013). Lack of metadata on historical land‐use change hinders the quantification of such impacts. Moreover, Slater *et al*. (2019) highlight that rivers are treated as conduits of fixed conveyance by models even though changes in channel geometry and structure are known to occur in response to periods of hydro‐climatic variability. Here, we assume that land‐use and channel geomorphology remain static over the period of reconstruction; a common assumption attached to long‐term flow reconstructions. Jones (1984) asserts that such assumptions can be justified. Water resource infrastructure designs are based on flows relating to current land use as opposed to historical conditions, suggesting that catchment response tuned to present conditions are a useful resource.

Second, potential biases or inaccuracies in precipitation data could propagate into the reconstructed flow series. The gridded Casty data set employed in this study was generated using both reanalysis and observed precipitation values, with principle component regression to interpolate across space. Interpolation of station data is more uncertain before the 1900s as the number of stations decreases rapidly prior to this time. Casty *et al*. (2005) highlight that European wide precipitation patterns in the early part of their series should be treated with caution, especially before 1800 when station numbers are low. For Ireland, we believe that data prior to 1850 should be treated with caution due to the sparseness of observed precipitation records on the island. A further source of uncertainty relates to the quality of early precipitation observations. Murphy *et al*. (2019) show that pre‐1870 winter precipitation observations in the United Kingdom were likely affected by under‐catch of snowfall due to gauge design and observer practice. It is likely that early Irish precipitation totals are affected by the same biases during winter months (Murphy *et al*., 2020).

Third, the sensitivity of hydrological model parameters to prevailing climatic conditions during the calibration period can result in uncertainties when models are used to simulate conditions different to those used for training. Broderick *et al*. (2016) showed that changes in climatic conditions can affect model performance depending on catchment, model type and assessment criteria. A shift from relatively wet to dry conditions resulted in poorer results. Future work should assess the robustness of monthly reconstructions to the wetness or dryness of periods used for training.

## 5 SUMMARY

This paper presents a data set of monthly river flow reconstructions back to 1766 for 51 Irish catchments. Gridded reconstructions of monthly precipitation and temperature, bias corrected to observed catchment data sets, are used with derived PET to force a conceptual hydrological model and an Artificial Neural Network to generate monthly flows spanning more than 250 years. Reconstructed flows are subject to uncertainties associated with hydrological response to arterial drainage and land‐use change, together with potential biases in early precipitation observations and non‐stationary hydrological model parameters. With these caveats in mind, the data set is suitable for examining hydrological responses to arterial drainage, tracking hydrological variability and change, or testing the robustness of water plans and/or contextualizing modern hydrological droughts.

### OPEN PRACTICES

This article has earned an Open Data badge for making publicly available the digitally‐shareable data necessary to reproduce the reported results. The data is available at https://doi.org/10.1594/PANGAEA.914306 Learn more about the Open Practices badges from the Center for OpenScience: https://osf.io/tvyxz/wiki.

## References

[gdj3107-bib-0001] Blaney, H.F. and Criddle, W.D. (1950) Determining water requirements in irrigated areas from climatological and irrigation data. Technical Paper No. 96, US Department of Agriculture, Soil Conservation Service, Washington, DC., USA, p. 48.

[gdj3107-bib-0002] Boé, J. , Terray, L. , Habets, F. and Martin, E. (2007) Statistical and dynamical downscaling of the Seine basin climate for hydro‐meteorological studies. International Journal of Climatology, 27(12), 1643–1655. 10.1002/joc.1602

[gdj3107-bib-0003] Brigode, P. , Brissette, F. , Nicault, A. , Perreault, L. , Kuentz, A. , Mathevet, T. *et al*. (2016) Streamflow variability over the 1881–2011 period in northern Québec: comparison of hydrological reconstructions based on tree rings and geopotential height field reanalysis. Climate of the Past, 12(9), 1785–1804. 10.5194/cp-12-1785-2016

[gdj3107-bib-0004] Broderick, C. , Matthews, T. , Wilby, R.L. , Bastola, S. and Murphy, C. (2016) Transferability of hydrological models and ensemble averaging methods between contrasting climatic periods. Water Resources Research, 52(10), 8343–8373. 10.1002/2016WR018850

[gdj3107-bib-0005] Broderick, C. , Murphy, C. , Wilby, R.L. , Matthews, T. , Prudhomme, C. and Adamson, M. (2019) Using a scenario‐neutral framework to avoid potential maladaptation to future flood risk. Water Resources Research, 55(2), 1079–1104. 10.1029/2018WR023623 31007298 PMC6472323

[gdj3107-bib-0006] Casty, C. , Handorf, D. and Sempf, M. (2005) Combined winter climate regimes over the North Atlantic/European sector 1766–2000. Geophysical Research Letters, 32(13). 10.1029/2005GL022431

[gdj3107-bib-0007] Casty, C. , Raible, C. , Stocker, T.F. , Wanner, H. and Luterbacher, J. (2007) A European pattern climatology 1766–2000. Climate Dynamics, 29, 791–805. 10.1007/s00382-007-0257-6

[gdj3107-bib-0008] Coron, L. , Thirel, G. , Delaigue, O. , Perrin, C. and Andréassian, V. (2017) The suite of lumped GR hydrological models in an R package. Environmental Modelling & Software, 94, 166–171. 10.1016/j.envsoft.2017.05.002

[gdj3107-bib-0009] Crooks, S.M. and Kay, A.L. (2015) Simulation of river flow in the Thames over 120 years: evidence of change in rainfall‐runoff response? Journal of Hydrology: Regional Studies, 4, 172–195. 10.1016/j.ejrh.2015.05.014

[gdj3107-bib-0010] Dastorani, M.T. , Moghadamnia, A. , Piri, J. and Rico‐Ramirez, M. (2010) Application of ANN and ANFIS models for reconstructing missing flow data. Environmental Monitoring and Assessment, 166(1–4), 421–434. 10.1007/s10661-009-1012-8 19543999

[gdj3107-bib-0011] Dawson, C.W. and Wilby, R. (1998) An artificial neural network approach to rainfall‐runoff modelling. Hydrological Sciences Journal, 43(1), 47–66. 10.1080/02626669809492102

[gdj3107-bib-0012] Dieppois, B. , Lawler, D.M. , Slonosky, V. , Massei, N. , Bigot, S. , Fournier, M. *et al*. (2016) Multidecadal climate variability over northern France during the past 500 years and its relation to large‐scale atmospheric circulation. International Journal of Climatology, 36(15), 4679–4696. 10.1002/joc.4660

[gdj3107-bib-0013] Fritsch, S. , Guenther, F. and Wright, M.N. (2019) Neuralnet: Training of neural networks, version 1.44.2., R package. Retrieved from CRAN.R‐project.org/package=neuralnet

[gdj3107-bib-0014] Gudmundsson, L. (2016) Qmap: Statistical transformations for post‐processing climate model output, version 1.0‐4, R package. Retrieved from CRAN.R‐project.org/package=qmap

[gdj3107-bib-0015] Gupta, H.V. , Kling, H. , Yilmaz, K.K. and Martinez, G.F. (2009) Decomposition of the mean squared error and NSE performance criteria: Implications for improving hydrological modelling. Journal of Hydrology, 377(1–2), 80–91. 10.1016/j.jhydrol.2009.08.003

[gdj3107-bib-0016] Hamon, W.R. (1961) Estimating potential evapotranspiration. Journal of Hydraulics Divisions, ASCE, 87, 107–120.

[gdj3107-bib-0017] Hanel, M. , Rakovec, O. , Markonis, Y. , Máca, P. , Samaniego, L. , Kyselý, J. *et al*. (2018) Revisiting the recent European droughts from a long‐term perspective. Scientific Reports, 8(1), 9499. 10.1038/s41598-018-27464-4 29934591 PMC6015036

[gdj3107-bib-0018] Harrigan, S. , Murphy, C. , Hall, J. , Wilby, R.L. and Sweeney, J. (2014) Attribution of detected changes in streamflow using multiple working hypotheses. Hydrology and Earth System Sciences, 18, 1935–1952. 10.5194/hess-18-1935-2014

[gdj3107-bib-0019] Jones, P.D. (1984) Riverflow reconstruction from precipitation data. Journal of Climatology, 4(2), 171–186. 10.1002/joc.3370040206

[gdj3107-bib-0020] Jones, P.D. , Lister, D.H. , Wilby, R.L. and Kostopoulou, E. (2006) Extended riverflow reconstructions for England and Wales, 1865–2002. International Journal of Climatology, 26(2), 219–231. 10.1002/joc.1252

[gdj3107-bib-0021] Kharrufa, N.S. (1985) Simplified equation for evapotranspiration in arid regions. Beiträge zur Hydrologie Sonderheft, 5(1), 39–47.

[gdj3107-bib-0022] Kling, H. , Fuchs, M. and Paulin, M. (2012) Runoff conditions in the upper Danube basin under an ensemble of climate change scenarios. Journal of Hydrology, 424, 264–277. 10.1016/j.jhydrol.2012.01.011

[gdj3107-bib-0023] Kuentz, A. , Mathevet, T. , Gailhard, J. , Perret, C. and Andréassian, V. (2013) Over 100 years of climatic and hydrologic variability of a Mediterranean and mountainous watershed: the Durance River. Cold and Mountain Region Hydrological Systems Under Climate Change: Towards Improved Projections, 360, 19–25.

[gdj3107-bib-0024] Lespinas, F. , Ludwig, W. and Heussner, S. (2014) Hydrological and climatic uncertainties associated with modeling the impact of climate change on water resources of small Mediterranean coastal rivers. Journal of Hydrology, 511, 403–422. 10.1016/j.jhydrol.2014.01.033

[gdj3107-bib-0025] Louvet, S. , Paturel, J.E. , Mahé, G. , Rouché, N. and Koité, M. (2016) Comparison of the spatiotemporal variability of rainfall from four different interpolation methods and impact on the result of GR2M hydrological modeling—case of Bani River in Mali West Africa. Theoretical and Applied Climatology, 123(1–2), 303–319. 10.1007/s00704-014-1357-y

[gdj3107-bib-0026] Machiwal, D. and Jha, M.K. (2006) Time series analysis of hydrologic data for water resources planning and management: a review. Journal of Hydrology and Hydromechanics, 54(3), 237–257.

[gdj3107-bib-0027] Maraun, D. (2016) Bias correcting climate change simulations‐a critical review. Current Climate Change Reports, 2(4), 211–220. 10.1007/s40641-016-0050-x

[gdj3107-bib-0028] McCarthy, G.D. , Gleeson, E. and Walsh, S. (2015) The influence of ocean variations on the climate of Ireland. Weather, 70(8), 242–245. 10.1002/wea.2543

[gdj3107-bib-0029] Mediero, L. , Kjeldsen, T.R. , Macdonald, N. , Kohnova, S. , Merz, B. , Vorogushyn, S. *et al*. (2015) Identification of coherent flood regions across Europe by using the longest streamflow records. Journal of Hydrology, 528, 341–360. 10.1016/j.jhydrol.2015.06.016

[gdj3107-bib-0030] Monteith, J.L. (1965) Evaporation and environment. Symposia of the society for Experimental Biology, 19, 205–234. 5321565

[gdj3107-bib-0031] Moravec, V. , Markonis, Y. , Rakovec, O. , Kumar, R. and Hanel, M. (2019) A 250‐year European drought inventory derived from ensemble hydrologic modeling. Geophysical Research Letters, 46(11), 5909–5917. 10.1029/2019GL082783

[gdj3107-bib-0032] Mouelhi, S. , Madani, K. and Lebdi, F. (2013) A structural overview through GR (s) models characteristics for better yearly runoff simulation. Open Journal of Modern Hydrology, 3(04), 179. 10.4236/ojmh.2013.34022

[gdj3107-bib-0033] Mouelhi, S. , Michel, C. , Perrin, C. and Andréassian, V. (2006) Stepwise development of a two‐parameter monthly water balance model. Journal of Hydrology, 318(1–4), 200–214. 10.1016/j.jhydrol.2005.06.014

[gdj3107-bib-0034] Murphy, C. , Harrigan, S. , Hall, J. and Wilby, R.L. (2013) Climate‐driven trends in mean and high flows from a network of reference stations in Ireland. Hydrological Sciences Journal, 58(4), 755–772. 10.1080/02626667.2013.782407

[gdj3107-bib-0035] Murphy, C. , Wilby, R.L. , Matthews, T. , Horvath, C. , Crampsie, A. , Ludlow, F. *et al*. (2020) The forgotten drought of 1765–1768: Reconstructing and re‐evaluating historical droughts in the British and Irish Isles. International Journal of Climatology, Early View(), 10.1002/joc.6521 PMC7818482 33519065

[gdj3107-bib-0036] Murphy, C. , Wilby, R.L. , Matthews, T. , Thorne, P. , Broderick, C. , Fealy, R. *et al*. (2019) Multi‐century trends to wetter winters and drier summers in the England and Wales precipitation series explained by observational and sampling bias in early records. International Journal of Climatology, 40(1), 610–619. 10.1002/joc.6208 32025091 PMC6988466

[gdj3107-bib-0037] Nash, J.E. and Sutcliffe, J.V. (1970) River flow forecasting through conceptual models part I‐A discussion of principles. Journal of Hydrology, 10(3), 282–290. 10.1016/0022-1694(70)90255-6

[gdj3107-bib-0038] Noone, S. , Broderick, C. , Duffy, C. , Matthews, T. , Wilby, R.L. and Murphy, C. (2017) A 250‐year drought catalogue for the island of Ireland (1765–2015). International Journal of Climatology, 37, 239–254. 10.1002/joc.4999

[gdj3107-bib-0039] Noone, S. and Murphy, C. (2020) Reconstruction of river flows and hydrological drought 1850‐2015. Climate and Society in Ireland: from prehistory to the present. Proceedings of the Royal Irish Academy, 120. In Press.

[gdj3107-bib-0040] Noone, S. , Murphy, C. , Coll, J. , Matthews, T. , Mullan, D. , Wilby, R.L. *et al*. (2016) Homogenization and analysis of an expanded long‐term monthly rainfall network for the Island of Ireland (1850–2010). International Journal of Climatology, 36(8), 2837–2853. 10.1002/joc.4522

[gdj3107-bib-0041] Oudin, L. , Hervieu, F. , Michel, C. , Perrin, C. , Andréassian, V. , Anctil, F. *et al*. (2005) Which potential evapotranspiration input for a lumped rainfall–runoff model? Part 2‐Towards a simple and efficient potential evapotranspiration model for rainfall–runoff modelling. Journal of Hydrology, 303(1–4), 290–306. 10.1016/j.jhydrol.2004.08.026

[gdj3107-bib-0042] Penman, H.L. (1948) Natural evaporation from open water, bare soil and grass. Proceedings of the Royal Society of London. Series A. Mathematical and Physical Sciences, 193(1032), 120–145. 10.1098/rspa.1948.0037 18865817

[gdj3107-bib-0043] Rudd, A.C. , Bell, V.A. and Kay, A.L. (2017) National‐scale analysis of simulated hydrological droughts (1891–2015). Journal of Hydrology, 550, 368–385. 10.1016/j.jhydrol.2017.05.018

[gdj3107-bib-0044] Slater, L.J. , Khouakhi, A. and Wilby, R.L. (2019) River channel conveyance capacity adjusts to modes of climate variability. Scientific Reports, 9(1), 1–10. 10.1038/s41598-019-48782-1 31477746 PMC6718627

[gdj3107-bib-0045] Smith, K.A. , Barker, L.J. , Tanguy, M. , Parry, S. , Harrigan, S. , Legg, T.P. *et al*. (2019) A multi‐objective ensemble approach to hydrological modelling in the UK: an application to historic drought reconstruction. Hydrology and Earth System Sciences, 23(8), 3247–3268. 10.5194/hess-23-3247-2019

[gdj3107-bib-0046] Spraggs, G. , Peaver, L. , Jones, P. and Ede, P. (2015) Re‐construction of historic drought in the Anglian Region (UK) over the period 1798–2010 and the implications for water resources and drought management. Journal of Hydrology, 526, 231–252. 10.1016/j.jhydrol.2015.01.015

[gdj3107-bib-0047] Thornthwaite, C.W. (1948) An approach toward a rational classification of climate. Geographical Review, 38(1), 55–94. 10.2307/210739

[gdj3107-bib-0048] Walsh, S. (2012) A summary of climate averages for Ireland 1981–2010. Climatological Note No. 15, Met Éireann, Glasnevin, Dublin.

[gdj3107-bib-0049] Wedgbrow, C. , Wilby, R.L. , Fox, H.R. and O'Hare, G. (2002) Prospects for seasonal river flow forecasting in England and Wales. International Journal of Climatology, 22, 217–236. 10.1002/joc.735

[gdj3107-bib-0050] Wilby, R.L. (2006) When and where might climate change be detectable in UK river flows? Geophysical Research Letters, 33, L19407. 10.1029/2006GL027552

[gdj3107-bib-0051] Wilby, R.L. , Clifford, N.J. , De Luca, P. , Harrigan, S.O. , Hillier, J.K. , Hodgkins, R. *et al*. (2017) The “dirty dozen” of freshwater science: Detecting then reconciling hydrological data biases and errors. WIREs Water, 4(3), e1209. 10.1002/wat2.1209

[gdj3107-bib-0052] Wilby, R.L. and Murphy, C. (2019) Decision‐making by water managers despite climate uncertainty. In: Pfeffer, W.T. , Smith, J.B. and Ebi, K.L. (Ed.) The Oxford Handbook of Planning for Climate Change Hazards. Oxford, UK Oxford University Press, pp. 1–34.

[gdj3107-bib-0053] Yan, B. , Fang, N.F. , Zhang, P.C. and Shi, Z.H. (2013) Impacts of land use change on watershed streamflow and sediment yield: An assessment using hydrologic modelling and partial least squares regression. Journal of Hydrology, 484, 26–37. 10.1016/j.jhydrol.2013.01.008

